# Similarities and Differences Between Patients Diagnosed with ANCA-Associated Vasculitis Who Are Positive and Negative for ANCA: University Clinic Practice and Expertise

**DOI:** 10.3390/medicina61081369

**Published:** 2025-07-29

**Authors:** Giedre Dereseviciene, Jolanta Dadoniene, Dalia Miltiniene

**Affiliations:** 1 State Research Institute Centre for Innovative Medicine, LT-08406 Vilnius, Lithuania; 2 Department of Public Health, Institute of Health Sciences, Faculty of Medicine, Vilnius University, LT-03101 Vilnius, Lithuania; 3 Orthopedics Traumatology and Reconstructive Surgery, Clinic of Rheumatology, Institute of Clinical Medicine, Faculty of Medicine, Vilnius University, LT-03101 Vilnius, Lithuania

**Keywords:** ANCA-associated vasculitis, ANCA-positive, ANCA-negative

## Abstract

*Background and objective.* Anti-neutrophil cytoplasmic antibody (ANCA)-associated vasculitis (AAV) affects small- to medium-sized vessels and is characterized by the production of ANCAs. The ANCA-negative term is used if the patient otherwise fulfills the definition for AAV but has negative results on serologic testing for ANCAs. The objective of this study was to compare ANCA-positive and -negative vasculitis patients and to evaluate the main differences possibly related to the presence of ANCAs. *Material and methods.* A cross-sectional study of 73 patients treated at the tertiary Rheumatology Centre of University Hospital from the 1 January, 2001, to the 31August, 2023, with diagnoses of AAV was carried out. Clinical characteristics and laboratory data were collected at the onset or at the first year of the disease. *Results.* Forty-eight (65.8%) patients were ANCA-positive, while twenty-five (34.3%) were ANCA-negative. Distribution by gender was similar in both groups, with a female–male ratio of 2:1. C-reactive protein (CRP) and erythrocyte sedimentation rate (ESR) were elevated for all AAV patients, but values were higher in the ANCA-positive patients’ group. The median hemoglobin was 106 g/L in the seropositive group and 127 g/L in the seronegative group. A higher prevalence of kidney involvement (60.4%) with elevated serum creatinine level (93.5 µmol/L) was observed in the ANCA-positive group compared with 24% and 70 µmol/l in the ANCA-negative group (*p* < 0.05). Neurological involvement was more frequently found in the ANCA-positive patient group, too: 29.2% compared to 20%. Among patients with ANCA-negative vasculitis, 88% had pulmonary; 92% ear, nose, throat (ENT); 48% joint; and 28% skin presentation. In comparison, involvement of these organs was less common in the ANCA-positive patients’ group, at 79.2%, 60.4%, 31.3%, and 25 %, respectively. *Conclusions.* ANCA-positive patients appear to be in a more difficult clinical situation in terms of organ involvement and laboratory changes.

## 1. Introduction

Small vessel vasculitis is a rare disease affecting predominantly small- to medium-sized vessels, resulting in occlusive, stenotic, or aneurysmal changes that lead to ischemic or hemorrhagic events [1]. Anti-neutrophil cytoplasmic antibody (ANCA)-associated vasculitis (AAV) affects small- to medium-sized vessels and is characterized by the production of antibodies specific for myeloperoxidase (MPO-ANCA) or proteinase-3 (PR3-ANCA). The ANCA-negative term is used if the patient otherwise fulfills the definition for AAV but has negative results on serologic testing for ANCAs. The main AAV clinicopathologic types are granulomatosis with polyangiitis (GPA), microscopic polyangiitis (MPA), and eosinophilic granulomatosis with polyangiitis (EGPA) [2]. PR3-ANCA is mostly associated with GPA, whereas MPO-ANCA is predominantly found in MPA patients. MPO-ANCA-positive vasculitis mainly affects lungs and kidneys, less frequently eyes, ear, nose, and throat, while PR3-ANCA-positive vasculitis is characterized by predominant involvement of the upper respiratory tract [3]. Miloslavsky et al. reported that ANCA-negative patients with a lower Birmingham Vasculitis Activity Score for GPA than PR3-ANCA-positive patients have a lower prevalence of renal involvement [4]. EGPA is commonly associated with asthma, rhinosinusitis, and peripheral eosinophilia. Although EGPA is classified as AAV, only 30% to 40% of cases are ANCA (mostly MPO)-positive [5]. Furthermore, geographic location and ethnicity influence differences in clinical presentations of patients with AAV, and the predominant ANCA type also varies geographically [6,7,8]. Overall, AAV commonly causes life-threatening organ damage, with a reported 5-year mortality rate of 28% and significant long-term morbidity in survivors [9]. Although more recent studies show a significant improvement in survival, with a 5-year survival rate of 94.5% [10], mortality in AAV patients is still 2.7-fold increased compared to the general population [11]. ANCA seropositivity is one of the factors influencing unachieved remission, relapse, renal involvement, and overall survival [12]. Additionally, ANCA positivity is often used in AAV diagnostic algorithms. In 2022, the American College of Rheumatology (ACR)/European League Against Rheumatism (EULAR) developed and approved new classification criteria that increased the weight of ANCA seropositivity [13,14,15]. On the other hand, although ANCA is helpful in diagnosing AAV and in selecting a homogeneous population for clinical trials, according to the revised 2017 international consensus on ANCA testing, the diagnosis of AAV should be based primarily on clinicopathological features [16]. Furthermore, using newer criteria with higher ANCA weighting, classification of two AAVs may occur [17]. The goal of this study was to describe the similarities and differences between ANCA-positive and -negative patients who were treated at the tertiary Rheumatology Centre of the University clinic.

## 2. Materials and Methods

A cross-sectional study of patients treated at the tertiary Rheumatology Centre of University Hospital from the 1 January 2001, to the 31 August 2023, with diagnoses of AAV was carried out. Data for the study were collected from electronic documents in the Electronic Medical Record of the University Hospital and from the documentation in the Rheumatology Center. The diagnoses were confirmed by clinical evaluation, serological ANCA testing, and histological analysis and fulfilled the requirements of the GPA, MPO, and EGPA nomenclature according to the revised criteria of the Chapel Hill consensus 2012 [2]. The ANCA-negative term was used if the patient otherwise met the definition for AAV but had negative results on serologic testing for ANCAs. Data on demographics (gender, age at the time of diagnosis), clinical characteristics (organ involvement), and laboratory data (C-reactive protein, erythrocyte sedimentation rate, hemoglobin level, white blood cell count, ANCA test result, serum creatinine level, and the growth of Staphylococcus aureus in the nasopharynx) was collected. ANCA positivity in the first patients enrolled in the study was determined by indirect immunofluorescence (IFL) that indicated c-ANCA or p-ANCA. Subsequently, enzyme-linked immunosorbent assays (ELISAs) showing anti-PR3 or anti-MPO ANCAs were also used together with IFL to determine ANCA positivity. Kidney involvement was defined as proteinuria, hematuria, elevated serum creatinine level, or by histopathological findings of pauci-immune necrotizing glomerulonephritis. Joint involvement was defined as arthralgia or arthritis, in some cases accompanied by myalgia. Lung or lower respiratory tract damage was determined if computed tomography (CT) or fiberoptic bronchoscopy (FBS) confirmed tracheal stenosis, bronchial stenosis, pleuritis, mucosal ulceration, granulomatous inflammation of the lower respiratory tract, signs of pulmonary hemorrhage, ground glass opacities, and/or foci in the lungs. Established bronchial asthma was also considered lung damage. Some cases of lower respiratory tract involvement were confirmed by histopathological findings of granulomatosis with polyangiitis or necrotizing granulomatous inflammation. ENT or upper respiratory tract involvement was assessed if rhinitis, sinusitis, otitis, epistaxis, hemoptysis, nasal septal ulcer, defect, perforation, or laryngeal stenosis was detected. Some cases of upper respiratory tract involvement were confirmed by histopathological findings of granulomatous or eosinophilic inflammation. Polyneuropathy or mononeuropathy was defined as neurological damage.

Clinical and laboratory data was collected at the onset or at the first year of the disease. We divided patients into two categories: seropositive (PR3-ANCA, c-ANCA, or MPO-ANCA, p-ANCA) and seronegative for ANCA. The patient was considered ANCA-positive if an ANCA-positive result was obtained at least once in cases where several tests were performed within the first year of diagnosis in order to rule out the influence of the immunosuppressive treatment on the ANCA positivity rate. Seroconversion later than 1 year from the date of diagnosis was not considered. For comparisons of numerical variables, we applied the Mann–Whitney U-test, and the Chi-square test was applied for associations of categorical variables. A *p* value < 0.05 was considered to be statistically significant. The study was approved by Vilnius Regional Bioethics Committee (approval number 158200-17-958-462). The study received a waiver for an informed consent form to be signed by participants.

## 3. Results

We analyzed 73 patients with a diagnosis of AAV: according to the 2012 Chapel Hill consensus criteria, GPA was diagnosed in 54.8% (40), MPA in 23.3% (17), and EGPA in 21.9% (16) of cases; 65.8% (48) had an ANCA-positive test, while in 34.3% (25) patients, ANCA was not detected. The distribution of ANCA-positive and ANCA-negative patients according to different AAV diagnoses was different in the study groups: according to the 2012 Chapel Hill consensus criteria, 48% (12) of ANCA-negative patients were diagnosed with GPA, 12% (3) with MPA, and 40% (10) with EGPA, while in the ANCA-positive group, 58.3% (28) of patients were diagnosed with GPA, 29.2% (14) with MPA, and only 12.5% (6) with EGPA. The distribution of study groups according to different AAV diagnoses and seropositive MPO-ANCA and PR3-ANCA patients in each AAV subgroup is shown in Table 1.

Considering the distribution of seropositive patients in each AAV disease group, PR3-ANCA was more prevalent in the GPA and EGPA groups, while MPO-ANCA was more prevalent in the MPA group

The mean age at the time of diagnosis was 48.8 years in the ANCA-positive group, similar to the ANCA-negative group—48.5 years, with MPA seronegative patients being the oldest at disease onset. Distribution by gender was similar in both groups, with a female-to-male ratio of 2:1. Interestingly, differences in the distribution of patients by gender were found in the AAV subgroups: all MPA-negative patients were female, while males were less likely to be seropositive than negative in the GPA group. The clinical and demographic characteristics of the studied groups are shown in Table 2 and Table 3.

The difference in kidney involvement was statistically significant between the groups, with higher incidence in the ANCA-positive group; 60.4% (29) of patients with positive ANCA serology had signs of kidney involvement, whereas only 24.0% (6) of ANCA-negative patients featured with kidney damage (*p*-value—0.0031). The difference was most significant in the GPA patient subgroup (*p*-value—0.0621). Renal involvement was confirmed by histopathological findings of pauci-immune necrotizing glomerulonephritis in 52.1% (25) of ANCA-positive patients, compared to 20% (5) of ANCA-negative patients. On the contrary, upper and lower respiratory tract involvement was present more often in the seronegative group: 92.0% (23) of patients had ear, nose, and throat (ENT) involvement and 88.0% (22) had pulmonary involvement, compared with 77.1% (37) and 85.4% (41) in the seropositive group. However, in the GPA subgroup, lung involvement was slightly more frequently found among ANCA-positive patients: 96.4% (27) compared to 91.7% (11). In the seronegative group of patients, airway stenosis was also found more frequently: 16% (4) compared to 6.25% (3). Furthermore, more patients with bronchial asthma were found in the ANCA-negative patient group: 28% (7) compared to 10.4% (5) in the ANCA-positive patient group, reflecting the more frequent diagnosis of EGPA in this group. Overall, respiratory histopathological changes supporting the diagnosis of AAV were found in 40% (10) of ANCA-negative patients and 31.25% (15) of ANCA-positive patients, confirming that respiratory lesions were more common in the seronegative patient group. Due to the relatively small sample size, statistical significance was not found; however, clinical difference was noted. There was no clinical and statistical significance noted for other organ involvement: 28.0% (7) of the patients in the ANCA-negative group had skin involvement, similar to 25.0% (12) in the ANCA-positive group. Arthralgia/arthritis was more frequent in ANCA-negative patients: 44.0% (11) compared to 39.6% (19) in the ANCA-positive group, but the result was not statistically significant, either. On the contrary, polyneuropathy or mononeuritis, assessed as a neurological system disorder, was more frequently found in the ANCA-positive patient group: 29.2% (14) compared to 20% (5), but the difference was not statistically significant. In the subgroup of GPA patients, damage to all assessed organ systems, except for ENT, was more often found among seropositive patients. The trend was not observed in the MPA and EGPA patient groups; on the contrary, skin involvement in both groups, ENT involvement in MPA patients, and joint damage in EGPA patients were more prevalent in the ANCA-negative group.

Laboratory indicators of inflammatory activity were elevated in both groups, with higher levels in the ANCA-positive group. The CRP median was 33.5 mg/L and ESR median 51.5 mm/h in the seropositive patient group compared to 21.0 mg/L and 42.5 mm/h in the seronegative group. Moreover, WBC was equally elevated in both the ANCA-positive and ANCA-negative groups: 9.1 × 10^9^/L and 9.6 × 10^9^/L, respectively. The difference in decreased hemoglobin levels, indicative of anemia, was statistically significant between groups. The median hemoglobin was 106 g/L in the seropositive group, in comparison to 127 g/L in the seronegative group. Furthermore, the serum creatinine level was higher in the ANCA-positive patients’ group, with a median of 93.5 µmol/L (minimum 33; maximum 1058) compared with a median of 70.0 µmol/L (minimum 58; maximum 493) in the ANCA-negative group; the difference was close to significance. Laboratory test results are also shown in Table 2.

## 4. Discussion

In 2022 ACR/EULAR developed and validated new classification criteria for GPA [13], MPA [14], and EGPA [15] that have improved sensitivity and specificity compared to previous criteria. According to the literature, the concordance rate between the new and previous criteria is 96.6% in the MPA group, 73.8% in the GPA, and 86.3% in the EGPA, with ANCA being the main discriminator [17]. However, newer criteria have been approved for the purpose of classifying vasculitis, which should be applied once the diagnosis is made to ensure that a homogeneous population is selected for clinical trials and research studies and that they are not appropriated for use in establishing a diagnosis of vasculitis [13].

This study was conducted to evaluate the similarities and differences between ANCA-positive and -negative patients with vasculitis, focusing on clinical characteristics and laboratory findings at the onset of the disease; therefore, we used the revised Chapel Hill consensus 2012 criteria for vasculitis nomenclature.

We found the most prominent differences in kidney damage were more likely in the ANCA-positive group, as well as lower hemoglobin levels found in this group. The patients were divided into two groups according to ANCA serology at the onset of the disease. Seroconversion was not taken into account. According to studies, either MPO-ANCAs or PR3-ANCAs are present in 90% of patients with GPA, MPA, or EGPA [18,19]; on the contrary, our cohort consists of 65.8% seropositive and 34.3% seronegative patients, indicating that more diagnoses were made based on clinicopathological features rather than a positive ANCA test. Although GPA was the most frequently detected AAV in both groups, accounting for 48% (12) in the ANCA-negative group of patients and 58.3% (28) in the ANCA-positive group of patients, there was a clear difference in the distribution of EGPA and MPA patients between the groups. In the group of seronegative patients, EGPA accounted for 40%, but in the group of seropositive patients, only 12.5%; on the contrary, MPA accounted for 12% and 29.2%, respectively. Our findings on the distribution of different AAVs in the groups support the findings of previous studies that patients diagnosed with EGPA are more likely to be ANCA-negative [5]. Overall, in our study, 37.5% of EGPA patients were ANCA-positive, while 70% of GPA patients and 82.4% of MPA patients were seropositive.

Although according to studies AAV is more common in men than in women, with a prevalence ratio between 1.07:1 and 1.48:1 and the peak incidence of vasculitis is at the age of 60–79 years [20,21], we estimated higher prevalence in women in both ANCA-positive and ANCA-negative groups: our estimated female-to-male prevalence ratio in both groups is 2:1. Moreover, we have found a younger age of the patients at the onset of the disease—48.8 years for ANCA-positive and 48.5 years for the ANCA-negative group. As shown by others and confirmed by our data, ANCA positivity is related to higher disease activity characterized by elevated inflammatory markers, decreased hemoglobin, and higher prevalence of renal involvement [4,9,12]. Furthermore, elevated baseline CRP at the onset of AAV disease has been observed to be predictive of renal damage and poor long-term outcome, a trend particularly associated with MPO-ANCA seropositivity [22]. We have found that laboratory inflammatory indicators CRP and ESR are elevated for all AAV patients, but values are higher in the ANCA-positive patients’ group. Due to the relatively small group, our estimated difference was not statistically significant. Higher prevalence of kidney involvement (60.4%) and elevated serum creatinine level (93.5 µmol/L) were observed in the ANCA-positive group, compared with 24.0% and 70.0 µmol/L in the ANCA-negative group.

On the contrary, ENT, pulmonary, skin, joint, and neurological involvement were not statistically different between the groups, confirming the results of previous studies and revealing that ANCA titers are more predictive of renal disease activity than nonrenal disease activity [23]. On the other hand, an inconspicuous difference in organ involvement between groups was established in our study. In our cohort, 88.0% of patients with ANCA-negative vasculitis had pulmonary, 92.0% ENT, 44.0% joint, and 28.0% skin presentation; in comparison, involvement of these organs was less common in the ANCA-positive patients’ group: 85.4%, 77.1%, 39.6%, and 25.0%, respectively. ANCA-positive patients were more likely to experience neurological symptoms, as polyneuropathy or mononeuropathy was observed in 29% of this group of patients compared with 20% of ANCA-negative patients. Analyzing separate disease subgroups, we have noted that in the GPA patients’ group, organ damage, except for ENT, was more prevalent among seropositive patients. The trend was not observed in MPA and EGPA patients’ groups, although, due to the small number of patients in each group, the statistical significance of the trend was not noted.

However, we did not take into account other organ involvement; AAV can involve any organ system, such as orbital structures, genitals, gastrointestinal, or cardiovascular [24].

AAV has complex symptomatology, and the specificity of ANCAs is limited. This might be explained by studies revealing that some ANCAs are more pathogenic than others, depending on the epitope specificity of the antibody [25]. Therefore, not the presence of ANCAs but their specificity might help predict relapse of AAV [26]. Furthermore, in some of these patients, laboratory tests for ANCAs can give false-negative results because of fragments of ceruloplasmin binding to the ANCAs in the serum [27].

This study focused on seropositivity rather than ANCA type. Moreover, since the first included patients were diagnosed with AAV as early as 2001, and the study analyzed only data from disease onset, ELISA testing was not available at that time; therefore, we do not have MPO and PR3 test results for some patients. This is a rather significant limitation of our study because, as mentioned in the literature, the ANCA type is closely related to the clinical presentation and prognosis, as patients with PR3-ANCA have more organs involved compared to patients with MPO-ANCA, resulting in a faster deterioration of renal function and more frequent relapses of the disease [26,28]. Moreover, a recent review of AAV relapse prognostic factors defined low- and high-risk relapse subgroups: women who are anti-PR-3 negative, with high creatinine levels and no ENT involvement, have a lower risk of exacerbation compared with men who are PR-3 positive, with low creatinine levels and ENT involvement [26]. On the other hand, some studies have revealed the opposite, that the type of disease (GPA or MPA) rather than the type of ANCA is more closely associated with relapse [4,29].

Furthermore, our study was limited by its single-center, retrospective character, based on hospital records where not every single clinical feature may have been recorded.

## 5. Conclusions

Our results support that diagnosis should be based on clinical features rather than ANCA assay results, as the differences in involvement of most organ systems between ANCA-positive and -negative patient groups were not statistically significant. However, ANCA-positive patients appear to be in a more difficult clinical situation in terms of kidney involvement and laboratory changes. Renal involvement should be at the forefront of clinical attention from the onset of the disease, followed by careful monitoring and management.

## Figures and Tables

**Table 1:** Distribution of ANCA-positive and ANCA-negative patients according to different AVV diagnoses.

	ANCA+			ANCA−
	MPO+ or p-ANCA	PR3+ or c-ANCA	Undifferentiated ANCA	
GPA according to 2012 Chapel Hill consensus criteria, *n* = 40 (%)	3 (7.5)	24 (60)	1 (2.5)	12 (30)
MPA according to 2012 Chapel Hill consensus criteria, *n* = 17 (%)	8 (47.1)	5 (29.4)	1 (5.9)	3 (17.6)
EGPA according to 2012 Chapel Hill consensus criteria, *n* = 16 (%)	2 (12.5)	4 (25)	.	10 (62.5)

ANCA—anti-neutrophil cytoplasmic antibody, AAV—ANCA-associated vasculitis, GPA—granulomatosis with polyangiitis, MPA—microscopic polyangiitis, EGPA—eosinophilic granulomatosis with polyangiitis.

**Table 2:** The descriptive statistics of clinical and demographic patients’ characteristics and laboratory test results at the onset of the disease.

	ANCA+ (PR3-ANCA, c-ANCA or MPO-ANCA, p-ANCA)	ANCA−	*p*-Value
Demographic characteristics			
Female, *n* (%)	32 (66.7)	17 (68.0)	
Male, *n* (%)	16 (33.3)	8 (32.0)	
Mean age at diagnosis (±SD)	48.8 (±11.9)	48.5 (±14.4)	
Median age at the time of diagnosis	51	48	
Clinical manifestation			
Pulmonary involvement*, n (%)*	41 (85.4)	22 (88.0)	0.7607
Kidney involvement *n* (%)	29 (60.4)	6 (24.0)	0.0031
Kidney biopsies performed *n* (%)	25 (52.1)	5 (20)	0.0082
ENT involvement *n* (%)	37 (77.1)	23 (92.0)	0.1139
Skin involvement *n* (%)	12 (25.0)	7 (28.0)	0.7816
Joint involvement *n* (%)	19 (39.6)	11 (44.0)	0.7150
St. aureus from the swab n (%)	7 (14.6)	4 (16.0)	0.8724
Neurological involvement *n* (%)	14 (29.2)	5 (20)	0.3970
Laboratory tests			
Serum CRP level, mg/L			0.3457
Median	33.5	21.0	
Minimum	0.3	0.2	
Maximum	279.0	310.0	
ESR, mm/h			0.96
Median	51.5	42.5	
Minimum	2	5	
Maximum	120	120	
WBC			0.5226
Average (±SD)	11.24 (±8.5)	10.23 (±3.9)	
Median	9.1	9.6	
Minimum	2.9	4.6	
Maximum	45.7	18.6	
Hemoglobin, g/L			0.00007
Average (±SD)	106 (±18.2)	127 (±19.9)	
Median	106	130	
Minimum	70	81	
Maximum	137	167	
Serum creatinine level, µmol/L			0.058
Median	93.5	70.0	
Minimum	33	58	
Maximum	1058	493	

ANCA—anti-neutrophil cytoplasmic antibody; ENT—ear, nose, throat; CRP—C-reactive protein; ESR—erythrocyte sedimentation rate; WBC—white blood cell count; SD—standard deviation.

**Table 3:** Differences between AAV subgroups based on ANCA seropositivity in terms of demographics and organ involvement.

	GPA		MPA		EGPA	
	ANCA+	ANCA−	ANCA+	ANCA−	ANCA+	ANCA−
Demographic characteristics						
Female, *n* (%)	20 (71.4)	7 (58.3)	8 (57.1)	3 (100)	4 (66.7)	7 (70)
Male, *n* (%)	8 (28.6)	5 (41.6)	6 (42.9)	0 (0)	2 (33.3)	3 (30)
Mean age at diagnosis	48.14	48.89	47.45	56.52	54.84	45.63
Median age at the time of diagnosis	51	48	51	49	50	48
Clinical manifestation						
Pulmonary involvement, *n* (%)	27 (96.4)	11 (91.7)	9 (64.3)	2 (66.7)	5 (83.3)	9 (90)
Kidney involvement, *n* (%)	16 (57.1)	3 (25)	13 (92.9)	3 (100)	0 (0)	0 (0)
ENT involvement, *n* (%)	26 (92.9)	12 (100)	6 (42.9)	3 (100)	5 (83.3)	8 (80)
Skin involvement, *n* (%)	7 (25)	0 (0)	4 (28.6)	2 (66.7)	1 (16.7)	5 (50)
Joint involvement, *n* (%)	12 (42.9)	4 (33.3)	5 (35.7)	1 (33.3)	2 (33.3)	6 (60)
Neurological involvement, *n* (%)	6 (21.4)	2 (16.7)	4 (28.6)	1 (33.3)	4 (66.7)	2 (20)

GPA—granulomatosis with polyangiitis, MPA—microscopic polyangiitis, EGPA—eosinophilic granulomatosis with polyangiitis.

## Data Availability

The participants of this study did not give written consent for their data to be shared publicly. This study received a waiver for an informed consent form to be signed by participants. The document granting permission to conduct the study without informed consent may be added upon request.
